# Is the presence of a catheter and time of surgery effective in conversion to open surgery in interval cholecystectomies after percutaneous drainage in acute cholecystitis?

**DOI:** 10.1590/1806-9282.20241051

**Published:** 2025-03-31

**Authors:** Gökhan Demiral, Ali Özdemir, Süleyman Kalcan, Hasan Gündoğdu, Ahmet Pergel

**Affiliations:** 1 Recep Tayyip Erdogan University, Faculty of Medicine, Department of General Surgery – Rize, Türkiye.; 2 Samsun University, Faculty of Medicine, Department of Radiology – Samsun, Türkiye.

**Keywords:** Gall bladder disease, Laparoscopic cholecystectomy, Acute cholecystitis, Conversion to open surgery

## Abstract

**OBJECTIVE:**

There are no guidelines regarding intraoperative or preoperative catheter removal in patients eligible for surgery following percutaneous drainage in acute cholecystitis. In this study, we evaluated the factors affecting the conversion to open surgery after percutaneous drainage and analyzed the relationship between catheter presence and time to operation in interval laparoscopic cholecystectomy.

**METHODS:**

In total, 50 patients with acute cholecystitis who underwent interval laparoscopic cholecystectomy after percutaneous drainage were retrospectively evaluated and grouped according to conversion to open surgery (Group 1) and non (Group 2). Factors that may be associated with conversion to open surgery and the presence of an intraoperative catheter were evaluated, and the time to surgery was calculated.

**RESULTS:**

There were 28 (56%) men and 22 (44%) women, and the mean age was 64 (±13) years. The severity of acute cholecystitis was moderate in 37 (74%) patients and severe in 13 (26%). When the groups were compared, no statistically significant difference was found between the presence of a catheter, the time of surgery 8 weeks before and after, and the conversion to open surgery. Postoperative hospitalization days were significantly higher in Group 1 (p=0.014).

**CONCLUSION:**

In patients who underwent interval laparoscopic cholecystectomy after insertion of a percutaneous drainage catheter in acute cholecystitis, the presence of a catheter and the waiting time for surgery after catheter insertion do not affect the rates of conversion to open surgery and complications.

## INTRODUCTION

Early laparoscopic cholecystectomy is the mainstay of treatment in acute cholecystitis (AcC)^1^. In high-risk patients who are not suitable for surgery, the clinical status of cholecystitis should be improved first.

In the TG18 guideline, it was revealed that appropriate treatment should be selected according to the severity grading of AcC, and a strategy was developed according to the severity of inflammation^2,3^. Antibiotherapy and percutaneous drainage (PD) have been recommended primarily to improve the clinically poor condition in moderate and severe cholecystitis in which surgery is postponed due to high morbidity and mortality.

Interval laparoscopic cholecystectomy (ILC) is performed in 28–67% of patients undergoing PD^4,5^. Fibrinous exudate and adhesion may be present due to the PD catheter, and this may exacerbate the existing AC adhesion. The effect of PD catheter on ILC in AcC and whether it positively or negatively affects surgical difficulty is unknown^2^. However, the questions of whether the PD catheter should be withdrawn before surgery and the optimal time of surgical intervention are controversial and still remain unclear^6^.

In this study, we evaluated the factors affecting the conversion to open surgery (COS) after PD and analyzed the relationship between catheter presence and time to operation in ILC.

## METHODS

This retrospective study was conducted between January 2018 and 2024. The data of patients who were clinically diagnosed with moderate to severe AcC according to TG-18^7^, hospitalized, and underwent PD by the Radiology Department were analyzed by evaluating the hospital information processing system. Approval was obtained from Recep Tayyip Erdoğan University Faculty of Medicine Clinical Research Ethics Committee (2024/149). Since this was a retrospective study, informed consent forms could not be obtained from the patients. A total of 160 patients with calculous AcC who underwent PD were evaluated, and 50 patients who underwent ILC were analyzed.

Patients with acalculous cholecystitis/cholangitis/pancreatitis, persistent biliary colic, choledocholithiasis, and concomitant malignancy were excluded. In addition, patients who underwent emergency surgery during the antibiotherapy phase after PD and patients who could not be operated on due to comorbidities after recovery, who did not want to be operated on, and who went to another health center during follow-up were also excluded.

Patients who underwent ILC were grouped as those who converted to open surgery (Group 1) and those who did not (Group 2). Age, gender, TG-18 cholecystitis grade, ASA score at the time of initial presentation, Charlson Comorbidity Index (CCI), C-reactive protein (CRP), white blood cell count (WBC), intensive care unit (ICU) hospitalization requirement, bile culture results at initial hospitalization, need for antibiotic change, need for catheter revision, emergency and elective surgery status, catheter withdrawal and operation time, medical problems after catheter withdrawal, and presence of PD catheter during surgery were recorded.

All patients with AcC were empirically started on intravenous antibiotics and fluid therapy for Gram-neagtive and -positive bacteria after diagnosis. PD catheterization was performed under local anesthesia, with the addition of sedoanalgesia, using the Seldinger method, US, and fluoroscopy, by placing a locked pigtail 8 French catheter transhepatically into the gallbladder. Medical treatment was organized according to the antibiogram in patients in whom bile cultures were sent. PD catheters were kept for at least 3 weeks. PD catheter was withdrawn intraoperatively in some patients; other patients were operated on after catheter withdrawal.

The research data were evaluated with SPSS v.23.0. Descriptive statistics of the groups were expressed as frequency and percentage (n, %). Differences in the distribution of categorical data between groups were evaluated by chi-square test. p<0.05 was accepted for significance.

## RESULTS

A total of 50 patients [28 (56%) males, 22 (44%) females] were included with a mean age of 64 (±13) years. The severity of AcC was moderate in 37 (74%) patients and severe in 13 (26%). Other basic characteristics during initial hospitalization and the subsequent period are given in Table 1.

**Table 1:** Basic characteristics of patients during initial hospitalization and the post-admission period.

		Mean±SD; n (%)
Age		64±13
Gender	Male	28 (56)
	Female	22 (44)
TG-18 degree of cholecystitis	Moderate	37 (74)
	Severe	13 (26)
ASA		2.75±0.7
	1	3 (6)
	2	10 (20)
	3	32 (64)
	4	5 (10)
CCI		3.75±2.18
First hospitalization CRP value		170±142
First hospitalization WBC value		14,514±6,066
First hospitalization, requirement for ICU admission	Yes	1 (2)
	No	49 (98)
First hospitalization, requirement for antibiotic change	Yes	8 (16)
	No	42 (84)
First hospitalization, bile culture positivity	Yes	17
	No	20
Presence of catheter during surgery	Yes	15 (30)
	No	35 (70)
Surgery time in patients operated on with a catheter: 58 days (30–95)	<8 weeks	8 (53.3)
	>8 weeks	7 (46.7)
Surgery time in patients operated on without catheter: 105 days (45–241)	<8 weeks	4 (11.4)
	>8 weeks	31 (88.6)
Interval cholecystectomy time in all patients: 91 days (30–241)	<8 weeks	12 (24)
	>8 weeks	38 (76)
Postoperative length of hospital stay		3.24±2.16 (2–14)
Total		50 (100)

SD: standard deviation, TG-18: Tokyo Guideline cholecystitis severity degree; CCI: Charlson Comorbidity Index, ASA: American Society of Anesthesiologists physical status classification; CRP: C-reactive protein, mg/dL; WBC: white blood cell count, 4,500–10,500/mm^3^; ICU: intensive care unit.

The variables and surgical outcomes of Groups 1 and 2 are compared in Table 2. When the patients were compared according to age (p=0.446), gender (p=0.179), TG-18 severity score (p=0.596), ASA/CCI score (p=0.485/0.145), CRP/WBC levels at initial hospitalization (p=0.107/0.369), ICU hospitalization requirements (p=0.78), antibiotic change requirement (p=0.57), culture status (p=0.612), and the presence of catheter during surgery (p=0.184), no significant difference was found between groups. The rates of COS were similar between the groups, depending on whether the patients had surgery before or after 8 weeks and the presence of a catheter. Postoperative hospitalization days were significantly longer in Group 1 (p=0.014) (Table 2).

**Table 2:** Statistical evaluation of the effect of patient variables on conversion to open surgery.

		Conversion to open surgery Mean±SD; n (%)		p
		Group 1 (Yes) 11 (22)	Group 2 (No) 39 (78)	
Age		66.64±14.5	62.8±12.61	0.446
Gender	Male	8 (28.6)	20 (71.4)	0.179
	Female	3 (13.6)	19 (86.4)	
TG-18 degree of cholecystitis	Moderate	8 (21.6)	29 (78.4)	0.596
	Severe	3 (23.1)	10 (76.9)	
ASA		2.91±0.94	2.69±0.61	0.485
	1	1 (33.3)	2 (66.7)	0.157
	2	2 (20)	8 (80)	
	3	5 (15.6)	27 (84.4)	
	4	3 (60)	2 (40)	
CCI		4.82±2.75	3.45±1.89	0.145
First hospitalization CRP value		123±103	189±149	0.107
First hospitalization WBC value		13,254±5,101	14,946±6,280	0.369
First hospitalization, requirement for ICU admission	Yes	0 (0)	1 (100)	0.78
	No	11 (22.4)	38 (77.6)	
First hospitalization, requirement for antibiotic change	Yes	2 (25)	6 (75)	0.57
	No	9 (21.4)	33 (78.6)	
First hospitalization, bile culture positivity	Yes	4 (23.5)	13 (76.5)	0.612
	No	5 (25)	15 (75)	
Presence of catheter during surgery	Yes	5 (33.3)	10 (66,7)	0.184
	No	6 (17.1)	29 (82.9)	
Surgery time in patients operated on with a catheter	<8 weeks	2 (25)	6 (75)	0.427
	>8 weeks	3 (42.8)	4 (57.2)	
Surgery time in patients operated on without catheter	<8 weeks	0 (0)	4 (100)	0.454
	>8 weeks	6 (19.3)	25 (80.7)	
Interval cholecystectomy time in all patients	<8 weeks	2 (16.6)	10 (83.4)	0.472
	>8 weeks	9 (23.7)	29 (76.3)	
Postoperative length of hospital stay		4.82±2.18	2.79±1.96	0.014
Total		11 (22)	39 (78)	

SD: standard deviation, TG-18: Tokyo Guideline cholecystitis severity degree; CCI: Charlson Comorbidity Index, ASA: American Society of Anesthesiologists physical status classification; CRP: C-reactive protein, mg/dL; WBC: white blood cell count, 4,500–10,500/mm^3^; ICU: intensive care unit.

Possible complications after PD and before and after ILK are given in Table 3. In the preoperative period, five patients were readmitted with cholecystitis and three with pancreatitis. Notably, one patient underwent ERCP for choledocholithiasis. In total, five patients underwent PD recatheterization. There were three patients in Group 1 who underwent only exploration and were operated on after 8 weeks without catheterization. Totally 19 patients were found to have severe adhesions causing intraoperative difficulties, and their numbers were similar in groups. Notably, one patient had a postoperative biliary fistula that resolved in the following days, and one patient had a hepatic flexure injury requiring reoperation and right hemicolectomy. A total of three patients developed pulmonary complications (one thromboembolism and two atelectasis effusion).

**Table 3:** Preoperative and intra-postoperative complications according to the conversion to open surgery, catheter status, and surgery time.

		Conversion to open surgery, n		Catheter status, n		Surgery time, n		Total, n
		Group 1	Group 2	Yes	No	<8 weeks	>8 weeks	
Preoperative complications	Cholecystitis	1	4	4	1	3	2	5
	Pancreatitis	1	2	0	3	1	2	3
	Choledocholithiasis (ERCP)	0	1	0	1	0	1	1
	Recatheterization	3	2	3	2	3	2	5
Intraoperative-postoperative complications	Only exploration	3	0	0	3	0	3	3
	Biliary fistula	0	1	0	1	0	1	1
	Adhesions	11	8	8	11	9	10	19
	Hepatic flexure fistula, right hemicolectomy	1	0	1	0	1	0	1
	Postoperative abscess	0	1	0	1	1	0	1
	Pulmonary thromboembolism	1	0	0	1	0	1	1
	Atelectasis, effusion	0	2	0	2	0	2	2

ERCP: endoscopic retrograde cholangiopancreatography.

## DISCUSSION

When we look at the studies in the literature on PD in AcC, it is observed that factors such as the effectiveness of the catheter in the early post-drainage period, when the catheter should be inserted, and the appropriate surgical time after catheterization are focused on. Kamezaki et al. reported that short-term (7–10 days) PD catheterization can be effective in AcC. This study focuses only on the solution of the problem in the acute period and does not include recommendations for the continuation and definitive solution of the problem, nor does it include the treatment approaches and surgical applications of the patients in the subsequent period^8^. Our study is the first to evaluate the effect of the presence of an intraoperative catheter on COS or surgical complications.

PD is an effective method that relieves symptoms related to biliary obstruction^9,10^. PD is considered sufficient for definitive treatment in elderly and surgically risky patients, whereas early or ILC is planned in relatively young patients whose morbidity improves after PD. After PTGBD, a fibrous tract appears at the catheter line that prevents bile leakage and reinfection. Once this tract is formed, the catheter can be safely removed. Recurrent cholecystitis and pancreatitis can occur after the catheter is removed, and if the catheter is left in longer than necessary, further adhesions can form, causing surgical difficulties and organ injuries. Furthermore, if the PD catheter is left in place for a prolonged time, quality of life may be negatively affected due to complications such as spontaneous catheter dislodgement, obstruction, and bile leakage through the skin^2,11^. In our study, five patients underwent recatheterization due to obstruction and dislodgement. In addition, nine patients required rehospitalization (five AcC, three acute pancreatitis, and one choledocholithiasis).

Although the controversy regarding the time of surgery in patients with PD continues, the majority of studies agree that the 8th week is the ideal time for ILC unless early surgery is performed after catheterization^6,12,13^. In a large study, LC complications were found to be higher in patients operated on early (within 1 month) after PTGBD, whereas PD catheter-related complications were higher in patients operated on late (after 8 weeks) and the ideal LC time was suggested to be between 4 and 8 weeks^14^. A Cochrane review showed that there was no significant difference in morbidity or mortality between patients who underwent early cholecystectomy within 96 h after PD and patients who underwent cholecystectomy after 8 weeks^15^. Altieri et al. reported that surgical complication risks and hospitalization time were higher in patients who preferred early cholecystectomy (<8 weeks) after PD^13^. In another study evaluating the efficacy of PD catheter, longer postoperative hospitalization and operation time, more blood loss, and a higher surgical difficulty score were reported in the AcC group with delayed LC compared to the non-PD group. It may be explained by fibrous adhesions formed by PD catheter^2^.

The data obtained in our study show that COS in ILC after PD is not associated with the presence of a catheter and operative times. Postoperative hospital stay was higher in the COS group, consistent with the literature. In a study of 132 patients examining the ideal time for cholecystectomy after PC, the rate of COS was 9.9%, and 25% of patients had mild cholecystitis^16^. In our study, the rate of COS was 22%. We thought that the fact that our study focused on patients with moderate and severe cholecystitis and excluded patients with mild cholecystitis by not performing PD was effective in this high rate.

There is no study in the literature on whether surgery after PD should be performed with or without a catheter. Surgeons who encounter difficulties in surgery due to the PD catheter hesitate about the choice of method. Patients waiting for ILC after catheter withdrawal may have recurrent cholecystitis and pancreatitis attacks and may require recatheterization, while surgery becomes more difficult in catheterized cases due to fibrosis caused by the PD catheter. Fibrosis and scarring caused by gallstone incarceration make surgery difficult. It has been shown that severe fibrotic changes and scarring persist in the Calot triangle even 8 weeks after PTGBD^2^.

It should be kept in mind that fibrosis and scarring that may occur due to PD catheter may be prevented by early LC after AcC. However, in high-risk patients with moderate and severe AcC who cannot tolerate urgent surgery in the acute period, PTGBD remains the first choice for the relief of pain and inflammation, and late ILC is often preferred for surgery in these patients. Since there is no general consensus, some patients are operated on in the presence of a PD catheter, while others are scheduled for surgery after catheter removal.

In our study, there were undesirable complications in both cases, including catheter-related fibrosis and adhesion of surrounding tissues, and catheter withdrawal-related cholecystitis, pancreatitis attacks, and the need for recatheterization. Cholecystitis, pancreatitis, the need for recatheterization, and adhesions causing difficulty in surgery were observed in similar numbers in all groups in accordance with the literature^16^. Additionally, biliary and hepatic flexure fistula were observed in one patient each, consistent with the literature.

On the other hand, we think that it is noteworthy that all of the patients in whom surgery was terminated with only exploration were operated on after 8 weeks and had no catheter, contrary to expectations. These results suggest that it may be more appropriate not to plan surgery after 8 weeks and support the previous study by Woodward et al.^14^. In the TG-18 guidelines, it is emphasized that if LC is to be preferred in moderate and severe cholecystitis, it is absolutely vital that the procedure be performed by surgeons with advanced skills. It has been stated that if the first medical facility consulted is not capable of providing full intensive care and treatments such as early cholecystectomy and biliary drainage, the patient should be quickly transferred to an appropriate center. During surgery, it should be noted that the degree of surgical difficulty may vary greatly depending on the level of inflammation and fibrosis, and rescue procedures, including COS, should not be avoided^3,17^. The fact that the complication rate in our study is lower than the studies in the literature shows the importance of having physicians experienced in advanced laparoscopic procedures and the necessary equipment.

This study has a few limitations. The study is observational and retrospective. Also, the number of patients is low. This could subject our findings to bias that would skew data results and conclusions. To confirm our results, studies with a larger number of cases and more comprehensive prospective investigations comparing the effects of the presence of a PD catheter would be useful.

## CONCLUSION

The presence of a catheter and the time of surgery do not affect the rates of COS in patients planned for ILC after PD. We recommend that the duration of surgery after PD should not be longer than 8 weeks. Particularly in patients with moderate and severe cholecystitis, the presence of a surgeon experienced in biliary tract and laparoscopic methods should be taken into account in surgical planning after PD, and the adequacy of technological facilities should be questioned.
